# A field trial of a fixed combination of permethrin and fipronil (Effitix^®^) for the treatment and prevention of flea infestation in dogs living with sheep

**DOI:** 10.1186/s13071-017-2145-1

**Published:** 2017-04-28

**Authors:** Manolis K. Chatzis, Dimitris Psemmas, Elias Papadopoulos, Christelle Navarro, Manolis N. Saridomichelakis

**Affiliations:** 1 Clinic of Medicine, Faculty of Veterinary Science, University of Thessaly, 224 Trikalon Str., GR-43132 Karditsa, Greece; 2 Laboratory of Parasitology and Parasitic Diseases, Faculty of Veterinary Medicine, Aristotle University of Thessaloniki, University Campus, GR-54124 Thessaloniki, Greece; 3 Virbac, 13ième-LID, 06511 Carros, France

**Keywords:** Canine, *Ctenocephalides canis*, *Ctenocephalides felis*, Fleas, Insecticide, Livestock

## Abstract

**Background:**

A large number of fleas parasitize dogs living with sheep in Greece. The primary aim of this randomized, blinded, placebo-controlled trial was to examine the efficacy of a permethrin-fipronil combination (Effitix^®^) for the treatment and prevention of flea infestation in dogs living with sheep and the secondary aim was to examine the efficacy of this intervention on flea infestation, pruritus and skin lesions of the people in contact with these dogs.

**Methods:**

Thirty dogs living with sheep and infested by at least 10 fleas and all 80 sheep living on the same premises were randomly allocated into equal groups. Group A dogs were treated three times, every 4 weeks, with a spot-on containing 54.5% permethrin and 6.1% fipronil, group A sheep were treated, on the same days, with a pour-on containing 1% deltamethrin, whereas group B dogs were sham-treated and group B sheep were placebo-treated. Flea counting was performed at the beginning of the trial (day 0) and after 14, 28, 56 and 84 days and the first five fleas from each animal were used for species identification. At the same time points, flea infestation, pruritus and skin lesions of the people in contact with the dogs were assessed.

**Results:**

The percentage of dogs with zero flea counts was significantly higher in group A than in group B on days 14, 28, 56 and 84 and flea counts were significantly lower in group A dogs than in group B dogs at the same time points. The percent efficacy of the permethrin-fipronil combination was higher than 78% (arithmetic means) or than 96% (geometric means) throughout the study. No adverse reactions were recorded. Between the two flea species found on dogs, *Ctenocephalides canis* was predominant over *C. felis*. Flea-infected sheep were not found at the beginning or during the study and no significant changes in flea infestation, pruritus and skin lesions of the people in contact with the dogs were witnessed throughout the study.

**Conclusions:**

A spot-on solution containing 54.5% permethrin and 6.1% fipronil is safe and effective for the treatment and prevention of *C. canis* and *C. felis* infestations in dogs living with sheep.

## Background

Fleas are common ectoparasites of dogs causing blood loss anemia, flea bite dermatitis and flea allergic dermatitis and transmitting parasites and bacteria of zoonotic importance [1–4]. Worldwide, *Ctenocephalides felis* is the most common flea species parasitizing dogs [2], but in Greece *C. canis* was more prevalent among dogs admitted to a University Teaching Hospital [5]. In addition, when dogs living in Greece on sheep and/or dairy goat farms were examined, the most common flea species found were *C. canis* and *Pulex irritans*, followed by *C. felis* [6, 7]. In previous studies, all dogs living with sheep had a high flea burden (median: 48 fleas per dog), the same was true for some of the sheep [7] and signs of severe flea infestation were witnessed in the people in contact with these dogs [6].

A commercially available spot-on solution containing a fixed combination of 54.5% permethrin and 6.1% fipronil (Effitix^®^; Virbac, France) is licensed for the treatment and prevention of flea and tick infestations and as a sand fly and mosquito repellent for dogs. Under laboratory conditions, this combination has been effective for the treatment of pre-existing and for the prevention of new infestations by *C. felis* for up to 30 days [8, 9] but, to the best of our knowledge, no studies on naturally infested dogs have been published. Also, a 1% deltamethrin pour-on solution (Deltanil^®^, Virbac, France) can be used for the treatment and prevention of various ectoparasites of sheep, including ticks, lice, keds and flies.

The primary aim of this randomized, blinded, placebo-controlled trial was to examine the efficacy of the fixed combination of 54.5% permethrin and 6.1% fipronil, when given every 4 weeks for three consecutive administrations, for the treatment and prevention of flea infestation in dogs living with sheep in Greece and the secondary aim was to examine the efficacy of this intervention on flea infestation, pruritus and skin lesions of the people in contact with these dogs.

## Methods

### Dogs

A total of 30 flea-infested dogs living with sheep in Greece were enrolled. The dogs included in the study should not present any abnormalities on physical examination (including skin lesions indicative of flea allergic dermatitis, like hypotrichosis-alopecia, excoriations, hyperpigmentation and lichenification in the posterior part of their body), they lived on the same premises with at least one more dog eligible for the study, had been infested by at least 10 fleas at the beginning of the trial (day 0) and they should not have been treated with ectoparasiticides, including pyrethroid-impregnated collars, for a minimum time period that was determined based on each product label. Dogs younger than 12 weeks of age, with a body weight of less than 1.5 kg, with known hypersensitivity to permethrin, fipronil or any of the excipients of Effitix^®^, as well as pregnant or lactating females were excluded.

**Fig. 1:**
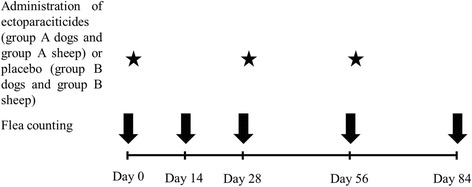
Design of the study. Time points of flea counting (*arrows*) and administration of the fixed combination of 54.5% permethrin and 6.1% fipronil (group A dogs), 1% deltamethrin (group A sheep), sham treatment (group B dogs) or placebo (group B sheep) (*stars*)

The dogs were randomly allocated into two groups (group A and group B). To this aim, all eligible dogs living on the same premises were considered as a block and per block randomization was done using a random number generator software, freely available on the internet (http://www.random.org/). Group A dogs (*n* = 15) were treated with the spot-on solution containing 54.5% permethrin and 6.1% fipronil (Effitix^®^), at the label dose (minimum dose: 60 mg/kg body weight permethrin plus 6.7 mg/kg body weight fipronil), every 4 weeks for three administrations (Fig. 1). The medication was applied directly onto the skin, on the dorsal midline, on two spots of approximately equal volume, one between the shoulders and one on the lumbar area, by one investigator (DP). Group B dogs (*n* = 15) were sham-treated with empty Effitix^®^ pipettes (due to non-availability of pipettes containing the excipients of the product) in the same way and at the same time intervals as in group A dogs (Fig. 1). The trial was conducted from the second half of May until the second half of December 2015 (from May to August for dogs living on premises A, B and C and from September to December for dogs living on premises D, E and F) and no other ectoparasiticides were used on the dogs or the environment.

Flea counts were performed, before treatment or sham-treatment, on days 0, 28, 56 and 84, as well as on day 14 (Fig. 1) by another investigator (MKC) who was blinded as to each dog’s group. Each dog was combed all over the body with a fine-tooth flea comb for at least 10 min and until no fleas could be recovered for the last 5 min and all live fleas were captured and counted [10]. The first five fleas were placed in 90% ethanol for species identification [11] and the remaining fleas were kept in a zip-closing plastic bag and were returned to the back of the dog at the end of the procedure.

In addition to the 1 h post-administration observation of all dogs by one investigator (DP), owners were instructed to monitor each animal for the duration of the study and to report any possible adverse events, whether considered to be treatment-related or not.

### Sheep

All 80 sheep living on the same premises with the enrolled dogs were eligible for the study provided that they were clinically healthy, lived on the same premises with at least one more eligible sheep, had not been treated with ectoparasiticides for at least 6 months and had no known hypersensitivity to deltamethrin or to any of the excipients of Deltanil^®^. Following the same randomization procedure used in dogs, sheep were treated with 1% deltamethrin pour-on solution (Deltanil^®^) at the label dose, every 4 weeks for three administrations (group A; *n* = 40) or with a pour-on solution containing the excipients of Deltanil^®^(group B; *n* = 40) at the same days as for the dogs (Fig. 1). Sheep were examined and flea counts were performed on the same days as for the dogs (Fig. 1). Initially, each sheep was inspected for 1 min for evidence of pruritus and then they were tipped up and combed with a fine-tooth flea comb, on the ventral trunk, from the genital area to the axillae (flea counting area) for at least 2 min and until no fleas could be recovered for the last minute. Monitoring for possible adverse events was the same as for the dogs.

### People in contact with the dogs

On days 0, 14, 28, 56 and 84, all people that were living and/or working on the premises were asked to self-assess their pruritus using a vertical 10 cm pruritus visual analogue scale [12] and to respond to the following questions: (i) “Have you seen fleas on your body for the last week?” (yes or no), (ii) “Is your pruritus less, the same or more severe than at the previous visit?”, and (iii) “Are your skin lesions due to fleas less, the same or more severe and/or extensive than at the previous visit?” (the last 2 questions were omitted at day 0).

### Statistical analysis

The sex, age and body weight of the dogs and the number of fleas per dog at day 0 were compared among the premises with Fisher’s exact test (sex) or one-way analysis of variance (ANOVA) (age, body weight, number of fleas). Also, the two groups of dogs were compared in terms of their distribution among the premises (Fisher’s exact test), sex (*χ* ^2^ test), age, body weight and number of fleas per dog on day 0 (independent samples *t*-test).

The geometric mean number of fleas for each group of dogs at the different time points of the study was calculated after adding 1 to the flea count of each dog, calculating the natural logarithm of (flea count +1), calculating the arithmetic mean of these logarithms, calculating the antilogarithm of the arithmetic mean and subtracting 1 from the antilogarithm. The percent efficacy of the 54.5% permethrin and 6.1% fipronil combination was calculated at the different time points of the study, using both the geometric and the arithmetic means of dog flea counts, with the formula $E=\frac{Mc- Mt}{Mc}\times 100$, where E is the percent efficacy, Mc is the mean (geometric or arithmetic) flea count in the controls (group B dogs) and Mt is the mean (geometric or arithmetic) flea count in dogs treated with the permethrin-fipronil combination (group A dogs).

The percentage of dogs with zero flea counts (primary outcome measure of the study) and the flea counts at the different time points of the study (secondary outcome measure) were compared between the two groups of dogs with Fisher’s exact test and with independent samples *t*-test, respectively. At a level of significance of 5%, the power of the study was 80% to detect a 54% difference between the two groups in the number of dogs with zero flea counts and it was 80% to detect a 21% difference of flea counts between the two groups of dogs, assuming a standard deviation of 0.2.

For each group of dogs separately, flea counts were compared among all time points of the study with Friedman’s two-way ANOVA; when a significant difference was found, *post-hoc* tests (related samples Wilcoxon Signed Rank test) were used to examine for differences between all pairs of time points.

The relative abundance of each flea species found on dogs was calculated as its percentage among all fleas identified, separately for group A and for group B dogs. The relative abundance was compared among the time points of the study with *χ* ^2^ or Fisher’s exact test.

The same statistical analysis employed for the dogs was planned for sheep but it was not performed because no flea-infested sheep were witnessed at time 0 and throughout the study (see Results).

The severity of pruritus of people in contact with the dogs, assessed by the visual analogue scale was compared among all time points of the study with Friedman’s two-way ANOVA; if a significant difference was found, *post-hoc* tests (related samples Wilcoxon Signed Rank test) were used to examine for differences between all pairs of time points.

Statistical analysis was done using SPSS 20 for Windows and the level of significance was set at 5%.

## Results

### Dogs, group allocation, treatment administration and adverse events

A total of 30 dogs, living on six different premises (designated as premises A, B, C, D, E and F) were screened and all of them were eligible for the study. The numbers of dogs for each of the premises are shown in Table 1. All dogs lived mainly outdoors and had free access to and close contact with sheep. Twenty-seven (90%) dogs were mongrels and 3 (10%) were English setters, 19 (63.3%) were intact males and 11 (36.7%) intact females, their age ranged from 1 to 6 years (mean ± standard deviation: 3.3 ± 1.6 years) and their body weight ranged from 5 to 35 kg (mean ± standard deviation: 20.3 ± 9 kg).

**Table 1:** Description of the six premises. Number of dogs and sheep in the six premises and allocation of the dogs into group A (54.5% permethrin and 6.1% fipronil-treated) and group B (sham-treated) and of sheep into group A (1% deltamethrin-treated) and group B (placebo-treated)

	Premise A	Premise B	Premise C	Premise D	Premise E	Premise F
Number of dogs	2	4	10	2	5	7
Group A dogs	1 (50.0%)	3 (75.0%)	7 (70.0%)	1 (50.0%)	1 (20.0%)	2 (28.6%)
Group B dogs	1 (50.0%)	1 (25.0%)	3 (30.0%)	1 (50.0%)	4 (80.0%)	5 (71.4%)
Number of sheep	20	16	20	4	8	12
Group A sheep	8 (40.0%)	10 (62.5%)	12 (60.0%)	1 (25.0%)	2 (25.0%)	7 (58.3%)
Group B sheep	12 (60.0%)	6 (37.5%)	8 (40.0%)	3 (75.0%)	6 (75.0%)	5 (41.7%)

Fifteen dogs were allocated to group A (54.5% permethrin and 6.1% fipronil) and 15 dogs were allocated to group B (sham-treatment) (Table 1). Flea counts at day 0 ranged from 18 to 69 fleas per dog (median: 29; arithmetic mean: 31.5; geometric mean: 29.6) in group A and from 18 to 48 fleas per dog (median: 28; arithmetic mean: 29.3; geometric mean: 28.3) in group B (Table 2). No significant differences were found in the distribution of the two groups of dogs among the six premises (*P* = 0.323) or in their sex (*P* = 0.705), age (*P* = 0.499), body weight (*P* = 0.552) and flea counts at day 0 (*P* = 0.582).

**Table 2:** Flea counts, number of dogs with zero flea counts and percent efficacy of 54.5% permethrin and 6.1% fipronil solution. Range, median, arithmetic and geometric means of flea counts of 54.5% permethrin and 6.1% fipronil-treated (group A) and of sham-treated (group B) dogs, number of group A and group B dogs with zero flea counts at the beginning of the trial (day 0) and after 14, 28, 56 and 84 days and percent efficacy of the 54.5% permethrin and 6.1% fipronil solution

	Day 0		Day 14		Day 28		Day 56		Day 84	
	Flea counts									
Group	A	B	A	B	A	B	A	B	A	B
Range	18–69	18–48	0–17	14–45	0–48	11–46	0–17	18–51	0–36	14–44
Median	29	28	0	33	1	35	0	32	0	28
Arithmetic mean	31.5	29.3	3.1	30.7	7.4	34.4	2.3	32.4	4.7	28.1
Geometric mean	29.6	28.3	0.8	29.4	1.3	32.7	0.7	31.5	1.0	26.9
	Number (%) of dogs with zero flea counts									
Group A (*n* = 15)	0 (0)		10 (71.4)		6 (42.9)		10 (71.4)		10 (71.4)	
Group B (*n* = 15)	0 (0)		0 (0)		0 (0)		0 (0)		0 (0)	
	Percent efficacy of 54.5% permethrin and 6.1% fipronil solution									
Arithmetic mean			89.8		78.6		92.9		83.3	
Geometric mean			97.1		96.1		97.8		96.6	

All treatments were administered according to the study protocol. The only deviation from the protocol was that the second visit to premise D was made with a 2 day delay (i.e. at day 16 instead of day 14). No adverse reactions were witnessed throughout the trial but one group A dog from premise C died due to a car accident between day 0 and day 14 (only day 0 data from this dog have been used in the statistical analysis).

### Flea counts of dogs and efficacy of the 54.5% permethrin and 6.1% fipronil solution

Range, median, arithmetic and geometric means of flea counts of group A and group B dogs at the various time points of the trial, the number of dogs with zero flea counts and the percent efficacy of the 54.5% permethrin-6.1% fipronil combination, based on both arithmetic and geometric means, are shown in Table 2.

The percentage of dogs with zero flea counts was significantly higher in group A than in group B at days 14 (*P* < 0.001, CI: 0.125–0.654), 28 (*P* = 0.006, CI: 0.363–0.899), 56 (*P* < 0.001, CI: 0.125–0.654) and 84 (*P* < 0.001, CI: 0.125–0.654) and flea counts were significantly lower in group A than in group B at days 14, 28, 56 and 84 (*t* < -5.64, *P* < 0.001 for all comparisons). In group A dogs, flea counts were significantly different among the five time points of the trial (*F* = 38.155, *P* < 0.001) and *post-hoc* testing revealed that they were significantly lower on days 14, 28, 56 and 84 compared to day 0 (*Z* < -3.234, *P* < 0.001 for all comparisons) with no difference between any other time points. On the contrary, no significant difference was found in flea counts of group B dogs among the five time points of the trial (*P* = 0.108).

The percent efficacy of the 54.5% permethrin-6.1% fipronil combination for the treatment of pre-existing and for the prevention of newly acquired flea infestations, under the conditions of this trial, was higher than 78% (arithmetic means) or higher than 96% (geometric means) on days 14, 28, 56 and 84 (Table 2).

### Species of fleas parasitizing dogs

Fleas were found on dogs of both groups on days 0, 14, 28, 56 and 84 and, with the exception of group A dogs on day 14, *C. canis* was predominant over *C. felis* (Table 3). In group A dogs, the relative abundance of *C. canis* was higher on day 0 compared to days 14 (*P* < 0.001, CI: 2.693–28.431) and 28 (*P* = 0.024, CI: 1.299–11.640) and on day 84 compared to day 14 (*χ* ^2^ = 4.375, *df* = 1, *P* = 0.036). In group B dogs, the relative abundance of *C. canis* was higher on day 56 compared to day 0 (*χ* ^2^ = 4.138, *df* = 1, *P* = 0.042).

**Table 3:** Flea species in group A and group B dogs. Relative abundance of each flea species on 54.5% permethrin and 6.1% fipronil-treated (group A) and sham-treated (group B) dogs at the beginning of the trial (day 0) and after 14, 28, 56 and 84 days

	Day 0	Day 14	Day 28	Day 56	Day 84
	Group A (54.5% permethrin and 6.1% fipronil solution)				
*Ctenocephalides canis*	70/79 (88.6%)	8/17 (47.1%)	16/24 (66.7%)	11/16 (68.8%)	16/20 (80.0%)
*Ctenocephalides felis*	9/79 (11.4%)	9/17 (52.9%)	8/24 (33.3%)	5/16 (31.3%)	4/20 (20.0%)
	Group B (sham treatment)				
*Ctenocephalides canis*	63/73 (86.3%)	65/73 (89.0%)	67/73 (91.8%)	70/73 (95.9%)	60/68 (88.2%)
*Ctenocephalides felis*	10/73 (13.7%)	8/73 (11.0%)	6/73 (8.2%)	3/73 (4.1%)	8/68 (11.8%)

### Sheep

Unexpectedly, no clinical signs of flea infestation and no fleas per flea counting area were seen on the 80 sheep on day 0 and throughout the trial. No adverse reactions were witnessed.

### People in contact with the dogs

A total of eight people lived and/or worked in the six premises (two in premises A and C and one in each of the remaining premises). Three of them (37.5%) had seen fleas on their body the week before time 0 and this figure remained constant throughout the trial. Although the mean severity of pruritus (Table 4) decreased during the trial, this change did not attain statistical significance (*P* = 0.112). However, none of these people reported increased severity of pruritus or increased severity and/or extend of flea-associated skin lesions compared to the previous visit at any time point of the study. On the contrary, decreased severity of pruritus and decreased severity and/or extend of flea-associated skin lesions on day 14 compared to day 0 were reported by two people, on day 56 compared to day 28 by one and on day 84 compared to day 56 by one.

**Table 4:** Severity of pruritus of people in contact with the dogs. Range, median and mean pruritus, assessed by a 10 cm visual analogue scale, of eight people living and/or working on the same premises where dogs were treated with either 54.5% permethrin-6.1% fipronil combination or were sham-treated and sheep were treated with either 1% deltamethrin or placebo at the beginning of the trial (day 0) and after 14, 28, 56 and 84 days

	Day 0	Day 14	Day 28	Day 56	Day 84
Range	0–10	0–1.3	0–0.9	0–0.1	0–0.1
Median	0	0	0	0	0
Mean	2.19	0.20	0.21	0.03	0.01

## Discussion

In this study, a spot-on solution containing 54.5% permethrin and 6.1% fipronil (Effitix^®^), when administered at the label dose three times at 4 week intervals, was effective for the treatment and prevention of flea infestation in dogs living with sheep. Indeed, at all time points after day 0, the percentage of dogs with zero flea counts (primary outcome measure) was significantly higher in treated compared to control dogs and flea counts (secondary outcome measure) were always higher in the latter. Furthermore, a significant decrease of flea counts on days 14, 28, 56 and 84 compared to day 0 was found in treated dogs, whereas no similar change was found in control dogs. Demonstration of efficacy under the conditions of this field trial may be of particular importance considering the high flea burden, as evidenced by the fact that all 30 screened dogs were eligible for the study (thus they were parasitized by at least 10 fleas) and by the median number of fleas per dog at time 0 (*n* = 28.5). Also, and in accordance with previous studies [13–15], the 54.5% permethrin and 6.1% fipronil spot-on solution was safe and no treatment-related side effects were witnessed.

The geometric mean-based percent efficacy (96.1–97.8%) of the 54.5% permethrin and 6.1% fipronil combination was lower compared to the 99.5–100% percent efficacy found in a previous laboratory study [8]. This may be explained by the following differences, regarding flea species and sources, environmental conditions and experimental design, between the two studies: (i) in the present study, dogs were naturally infested, mainly by *C. canis* and secondarily by *C. felis*, whereas a laboratory strain of *C. felis* was used in the previous investigation. Therefore, it is possible that the laboratory strain may have been more susceptible to the active ingredients of Effitix^®^ compared to the field strains of fleas infesting dogs in the present study; (ii) in the previous study, dogs were housed indoors, under controlled environmental conditions and were not exposed to rain, whereas in the present study dogs lived mainly outdoors, under variable environmental conditions and may have repeated exposure to rain. Indeed, raining was recorded in the area during 21 (premise C), 27 (premises A and B) or 43 (premises D, E and F) out of the 84 days of the trial (data from http://www.meteo.noa.gr/index.html). The lack of influence of 2–3 immersions in water during a 30 day period on the efficacy of another spot-on containing 50.48% permethrin and 6.76% fipronil (Frontline Tri-Act^®^/Frontect^®^; Merial) [16] does not negate this possibility due to differences in the excipients and the concentration of active ingredients between the two products and due to the different frequency of hair coat wetting (6.7–10% of the 30 days of the previous laboratory study *vs* up to 25–51.2% of the 84 days of our field study, assuming that dogs were not sheltered during rain); (iii) in the laboratory study on the efficacy of Effitix^®^ against *C. felis*, treated and control dogs were kept separately [8] whereas close contact among them was unavoidable in the present study and it is possible that passive transfer of Effitix^®^ active ingredients from treated to control dogs may have occurred. However, the absence of a significant reduction in the flea counts of sham-treated dogs is not in favor of this explanation; (iv) in the laboratory study, flea counting was performed 24 or 48 h after experimental infestation, whereas in the present study dogs were constantly exposed to newly emerging fleas. Although the speed of the anti-flea action of Effitix^®^ is not published, data on the 50.48% permethrin and 6.76% fipronil combination spot-on (Frontline Tri-Act^®^/Frontect^®^; Merial) show a geometric mean-based percent efficacy against *C. canis* at 1–6 h post-infestation of 92–100% at 14 days and of 55.7–99.1% at 28 days [17] and an arithmetic mean-based percent efficacy against *C. felis* at 1–12 h post-infestation of 96.2–100% at 14 days and of 73.4–99.6% at 28 days [18]. Therefore, it is probable that the fleas we found on group A dogs on days 14, 28, 56 and 84 represented new infestations by fleas from the environment or from group B dogs.

The arithmetic mean-based efficacy (78.6–92.9%) was numerically lower compared to the geometric mean-based efficacy (96.1–97.8%) of the 54.5% permethrin and 6.1% fipronil combination throughout this study. The low arithmetic mean-based efficacy occurred mainly due to the relatively high flea counts of two group A dogs (both from premise B) that were parasitized by 9–39 and by 2–48 fleas, respectively, at all time points after day 0 (data not shown). Numerous factors may explain the low efficacy of Effitix^®^ in these two dogs, including improper application of the product, individual differences in the diffusion of permethrin and fipronil across the skin and in their concentration in the epidermal and/or sebaceous gland lipids [19], higher exposure to rain and increased exposure to newly emerging fleas before flea counting. Nevertheless, these results confirm that the geometric mean-based efficacy is a superior measure of central tendency compared to the arithmetic mean-based efficacy [17].

Approximately one year earlier (from April to July 2014), we had conducted a similarly-designed randomized, blinded, placebo-controlled trial of 3 month-duration, to examine the efficacy of spinosad (Comfortis^®^; Elanco Animal Health) for the treatment and prevention of flea infestation in dogs living with sheep [7]. In that study, the geometric-mean based efficacy of spinosad ranged from 99.4 to 100%, the arithmetic-mean based efficacy ranged from 98.7 to 100% and the percentage of dogs with zero flea counts ranged from 80 to 100%. Besides differences in the efficacy between the two products, there are many other possible explanations for these results: (i) the two studies were conducted on different years and they did not include the same dogs or even the same premises; (ii) the flea species and their relative abundance were different. Although dogs were infested by the same two flea species on day 0, the relative abundance of *C. canis* was numerically lower in the present (88.6%) compared to the previous study. Also, *P. irritans* infestation was not witnessed in the present study whereas it was the predominant flea species on placebo-treated dogs from day 14 until the end (day 84) of the previous study; (iii) in the present study, flea combing was continued until no fleas could be recovered for the last 5 min whereas in the previous study it was continued until no fleas could be recovered for 1 min; (iv) due to its systemic mode of action, the efficacy of spinosad is not expected to be negatively influenced by environmental factors, such as exposure to rain; (v) passive transfer of spinosad from treated to control dogs is not expected to occur; and (vi) in the present study flea combing was performed in the premises whereas in the previous study dogs were separated from the sheep and removed from the heavily-infested area for at least 4 h before flea counting, thus minimizing the chances of newly-acquired flea infestations [7].

The relative abundance of *C. canis* over *C. felis* was inversed in group A dogs 14 days after the first application of the 54.5% permethrin and 6.1% fipronil spot-on solution and remained significantly lower on day 28 compared to day 0, to increase afterwards to pre-treatment levels. This may imply that Effitix^®^ can control *C. canis* infestations in a shorter time frame compared to *C. felis* and further studies are needed to prove or reject this hypothesis. Similarly, the relative abundance of *C. canis* over *C. felis*, although not inversed, was significantly lower on day 14 compared to day 0 in spinosad-treated dogs living with sheep [7].

In our previous investigation, *P. irritans* was the predominant flea species found on sheep at all time points and the predominant flea species found on placebo-treated dogs on days 14, 28, 56 and 84 [7]. Its absence in the present study should be attributed to the widespread use of ectoparasiticides and/or insect repellents on sheep, during winter 2014–2015, due to an outbreak bluetongue disease. Although sheep had not been treated for at least 6 months before enrollment (at least according to their owners), previous interventions may have reduced *P. irritians* populations to the degree that none of the sheep presented clinical signs of flea infestation and all of them had zero flea counts per flea counting area at the beginning and throughout the trial. Nevertheless, finding no fleas when combing the ventral trunk of the sheep for 2 min does not necessarily imply that sheep were free of fleas and finding no *P. irritants* on dogs does not preclude the possibility of its presence in low numbers, given that only 5 fleas from each dog were used for species identification.

Workers in dairy goat and sheep farms in Greece are frequently infested by fleas [6]. According to the “One Health” concept, an effective therapeutic and/or preventive regimen against flea infestation in farm animals and dogs living in close proximity to them should also control human infestation. In the present study, the prevalence of flea infestation of people in contact with the dogs remained constant (37.5%) throughout the trial which underlines that the control of human infestation probably necessitates treatment of all animal reservoirs and of infested premises. On the other hand, some people reported that the severity of their pruritus and the severity and/or extend of their flea-associated skin lesions were reduced and at the same time a non-significant (probably due to the low statistical power) reduction of pruritus assessed by the visual analogue scale was witnessed. Despite lack of a control group, these observations may imply that effective flea control, even in half of the dogs (and half of the sheep), may contribute to a reduction of flea-induced clinical signs of the people in contact with these dogs.

## Conclusions

When administered every 4 weeks for three times in dogs living with sheep, the efficacy of a 54.5% permethrin and 6.1% fipronil spot-on solution (Effitix^®^) for the treatment and prevention of infestations by *C. canis* and *C. felis* was ≥ 78.6% (arithmetic means) or ≥ 96.1% (geometric means). The efficacy of this solution is further supported by the significantly higher number of treated dogs with zero flea counts (≥ 71.4%) compared to the sham-treated group. The results demonstrate that the 54.5% permethrin and 6.1% fipronil spot-on solution is safe and effective for the treatment and prevention of flea infestation in dogs living with sheep.

## Abbreviations

ANOVA: Analysis of variance
